# Socio-demographic effects on role assignment and associated occupational health and safety issues in artisanal and small-scale gold mining in Amansie Central District, Ghana

**DOI:** 10.1016/j.heliyon.2023.e13741

**Published:** 2023-02-14

**Authors:** Peter Donkor, Ebenezer Kwadwo Siabi, Kwasi Frimpong, Samuel Kofi Mensah, Elikplim Sarah Siabi, Christopher Vuu

**Affiliations:** a School of Public Service and Governance, Ghana Institute of Management and Public Administration, P. O. Box AH50, Achimota, Accra, Ghana; b Earth Observation Research and Innovation Center (EORIC), University of Energy and Natural Resources, P. O. Box 214, Sunyani, Ghana; c Department of Civil and Environmental Engineering, University of Energy and Natural Resources, P. O. Box 214, Sunyani, Ghana; d Department of Civil Engineering, College of Engineering, KNUST, Kumasi-Ghana/ Regional Water and Environmental Sanitation Centre, Kumasi (RWESCK), KNUST, Ghana

**Keywords:** Safety, Mining, Occupational hazards, Occupational health, Occupational accidents, Risk factors, OHS, Occupational Safety and Health, ASGM, Artisanal and small-scale gold mining, EPA, Environmental Protection Agency, MinCom, Minerals Commission, MDAs, Municipal and Districts Assemblies, PPE, Personal Protective Equipment, COR, Crude Odd Ratios, AOR, Adjusted Odd Ratios, LI, Legislative Instrument

## Abstract

The study employed the binary logistic regression model, Chi-square, and likelihood ratio test to explore the nexus between socio-demographic characteristics and role assignment as well as Occupational Health and Safety (OHS) issues in artisanal and small-scale gold mining (ASGM) undertakings in the Amansie Central District of Ghana. Simple random sampling was employed to sample 250 respondents from three (3) different mining sites. The results revealed that socio-demographic variables such as age, gender, and work experience significantly impacted the type of role assumed by individuals in ASGM undertakings. For the socio-demographic effects on OHS issues, male respondents especially those in the age group between 18 and 35 with less work experience and education had a higher risk of recording injuries/accidents. Other risk factors such as types of role, reasons for ASGM, awareness of OHS hazards, awareness of Personal Protective Equipment (PPE), usage of PPE, arrest for not using PPE, cost of PPE, and frequency of buying PPE had a statistically significant influence on the occurrence of injuries/accidents. It is recommended that the Government implement initiatives to ensure that workers in ASGM operations in Ghana are provided with training, education, resources, and support services to ensure their safety and well-being, taking into account their socio-demographic characteristics. Also, the government and related stakeholders create more jobs through sustainable mining with long-term potential in local districts to address goals 1 (No poverty) and 2 (zero hunger) of the sustainable development goals.

## 1 Introduction

Artisanal and small-scale gold mining (ASGM) is a widespread activity in many low and middle-income countries, particularly in sub-Saharan Africa [[1], [2], [3], [4], [5]]. It has been estimated that over 10 million people in over 70 countries depend on ASGM for their livelihoods [6]. In Ghana, ASGM contributes significantly to the country’s gold production and accounts for about one-third of total gold production [7,8]. Despite its economic importance, ASGM is associated with several environmental, health, and safety risks, including occupational health and safety (OHS) issues [[9], [10], [11], [12]].

The sector is largely informal [[13], [14], [15], [16], [17]] and often associated with poverty, environmental degradation, and health and safety risks [[18], [19], [20]]. The assignment of roles in ASGM is determined by a range of factors, including socio-demographic characteristics. The socio-demographic characteristics of ASGM workers, such as age, gender, work experience, and education, have been identified as important factors influencing the decision-making process, the roles and responsibilities that individuals assume in the sector, and the associated OHS risks [4]. Evidence from research indicates that the role of individuals in ASGM is strongly influenced by socio-demographic characteristics. For example, certain roles may be restricted to certain genders or ages, or certain ethnicities may be given preferential treatment in terms of access to resources [13,21].

The study aimed at exploring the Socio-demographic effects on role assignment in ASGM in Amansie Central District, Ghana. The study of ASGM has become more important due to the recent increasing recognition of the sector’s contribution to global gold production and the potential for the sector to make a positive contribution to socio-economic development in developing countries. Furthermore, there is an urgent need to address the environmental, health, and safety risks associated with ASGM activities. As such, the findings of the study are expected to provide a valuable contribution to the understanding of the socio-demographic characteristics of the assignment of roles and its associated OHS issues in the ASGM sector. Thus, the paper contributes to the existing body of knowledge by exploring the Socio-demographic effects on role assignment in ASGM in Ghana. Moreover, the findings of this study will help inform policy and practice in the sector which takes into account the socio-demographic characteristics of workers, particularly in terms of improving OHS.

## 2 Methods and materials

### 2.1 The study area

**Fig. 1:**
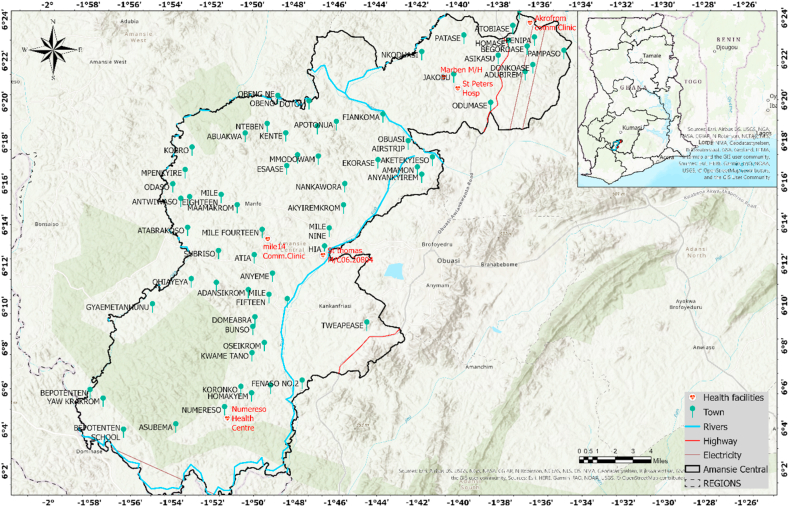
Map of the studied area.

Generally, the study was undertaken in Ghana’s Ashanti Region where the Amansie Central District is located. Largely, the district is bordered by the following areas: Adansi North, Amansie West, Upper Denkyira district, Adansi South, Bekwai Municipality, and the Obuasi Municipality (see Fig. 1) [22,23]. The area is bounded by Longitudes 1°00 W and 2°00 W and Latitudes 6°00 N and 6°30 N. Geological formation of the district span the Tarkwaian, Granite rocks, and the Birimian—with abundant deposits of minerals such as gold [22,24]. Significantly, the district has a total landmass of 710 km^2^ with a human population of 110,026 [22,25]. The area roughly has 220 settlements or communities, with Jacobu serving as the district's capital [22]. The district has a climate of the semi-equatorial type while topographically, has moderately flat with a fairly undulating plateau [22]. Major tree species (e.g., mahogany, Wawa, Sapele, Odum, Edinam, etc) are located in the area. The locality has a bi-modal rainfall variability, with the major season lasting from March to July and the minor season lasting from September to November—drained largely by the two major rivers, Oda and Offin [22]. Other minor rivers equally exist [23]. The district has two major forest reserves covering Subin and Oda [22] with vegetation particularly depicting the semi-deciduous and rainy forest types. Due to this, the terrain is exceptionally fertile and well-suited for agriculture [22,23]. Also, due largely to the locality’s huge deposit of gold as an invaluable mineral, has attracted legal and largely, illicit ASGM undertakings. While some of these firms are granted either a permit or/licenses to undertake prospecting works regarding a sizable concession—the majority of illegal ASGM operators equally take advantage of the situation to illicitly engage in ASGM. The majority of these ASGM miners mostly resort to rudimentary techniques of extracting and processing gold with little or no care for the environment coupled with the health and safety of the miners themselves (see e.g., Refs. [9,10,26]).

### 2.2 Research design, data collection, and analysis

The study generally utilized the cross-sectional descriptive survey. The respondents for the study were sampled using purposive and simple random sampling methods. A descriptive research design together with a field survey focused on socio-demographic characteristics of respondents and OHS issues were employed. Players of ASGM who freely accepted to contribute to the study were engaged. Simple random sampling was employed to pick 250 respondents from three (3) different mining sites using Sloven’s formula (Eqn (1)). The selected sample size for each site is shown in Table 1.

(1) $$Sample\;Size=\frac{N}{1+{\left(Ne\right)}^{2}}$$

Where:

**Table 1:** Sample size for each mining site.

Mining site	Population	sample
Site 1	150	109
Site 2	110	86
Site 3	64	55

N = population size, e = error margin or tolerance of 5%

To obtain data from key informants, the study used key informant interviews and field observations as well as semi-structured questionnaires. An Officer from the Minerals Commission (MinCom) and the Environmental Protection Agency (EPA) respectively was purposively sampled as the key informants for the study—These officers were purposively sampled. For this reason, 252 respondents were used for the survey. Before exploring the mining sites, The Enforcement and Compliance Monitoring Department of EPA, Obuasi Area Office, Obuasi, gave its ethical permission before the survey was carried out at the gold-mining locations. Subsequently, a presentation was done to explain the main and specific objectives of the study, and the consent of respondents was sought. Respondents encompassed those who willingly accepted to participate.

The study employed questionnaires to solicit data on the socio-demographic characteristics of respondents and the type of roles of respondents in the ASGM. Moreover, the effects of Personal Protective Equipment (PPE) use and the occurrence of injuries/accidents with ASGM were assessed. Also, data on the basic OHS concerns for OHS were collected. There were two types of questions on the questionnaires: open-ended and closed-ended questions. Respondents were asked to choose the most appropriate response that expressed their opinions from a list of alternative responses to the closed-ended questions. However, the closed-ended questions were without alternative responses. Moreover, the Likert scale [27] with a range of one (1) to five (5) was used to score the questionnaire responses. The study employed self-administered questionnaires.

Generally, the English language was used in the administration of questionnaires, however, where respondents were unable to understand, a trained enumerator was available to administer the questionnaire in a widely used local dialect (Twi). Ample time was allowed for respondents to go over the questions thoroughly before answering to guarantee that they offered suitable responses and addressed questions in a manner they comprehended them. Before proceeding with the entire administration of the questions, pretesting was done to ensure that the questionnaires were in good working order. To guarantee the reliability and consistency of the data obtained, sequentially, the interview guides and questionnaires were numbered. The anonymity and confidentiality of respondents were protected; thus, they were under no need to disclose information. The gathered data was examined using the SPSS 25 software. Employing SPSS, the fundamental propensity and some factors, including the standard deviations of the various variables, were determined. A bar chart was used to illustrate the results and observations. Tables, bar charts, and frequency distribution were used to illustrate the data.

The binary logistic Regression analysis was used to explore the association between the independent variables (Socio-demographic features of respondents such as gender, age, work experience and education level, awareness of occupational health hazards in ASGM, Awareness of PPE, usage of PPE, arrest for not using PPE, cost of PPE, Frequency of buying PPE) and dependent variables (the type of roles in ASGM, reason for ASGM and the occurrence of injuries/accidents). Individual variables served as input for the binary logistic regression model and were categorized with a significance level of less than 0.05 noted. The evaluation of individual variables against the dichotomized socio-demographic features, reason for ASGM, and occurrence of injuries/accidents generated the crude odd ratios (COR) as well as a confidence interval of 95%. All the variables further served as input for the multivariate binary logistic regression model to generate the adjusted odd ratios (AOR) with varying confidence intervals.

Therefore, the study tested the following hypotheses using the Pearson Chi-square and likelihood ratio test as well as the binary logistic regression model.

1. H_0_ = Gender influences the assignment of roles in ASGM

H_1_ = Gender does not influence the assignment of roles in ASGM.

2. H_0_ = Age influences the assignment of roles in ASGM

H_1_ = Age does not influence the assignment of roles in ASGM.

3. H_0_ = Education influences the assignment of roles in ASGM

H_1_ = Education does not influence the assignment of roles in ASGM.

4. H_0_ = work experience influences the assignment of roles in ASGM

H_1_ = work experience does not influence the assignment of roles in ASGM.

5. H_0_ = Risk factors have an influence on the occurrence of injuries/accidents in ASGM
6. H_1_ = Risk factors have no influence on the occurrence of injuries/accidents in ASGM

The relationship between the variables is statistically significant if the p-value is equal to or less than alpha level (α = 0.01, 0.05, and 0.1).

### 2.3 Evaluation of model accuracy

Depending on the type of model adopted in logistic regression, there are different methods of evaluating the observed and fitted values [28]. One of the commonly applied methods for the goodness-for-fit test in binary logistic regression is the Hosmer and Lemeshow test [28]. This test is in-built into the SPSS package and is generally accepted [29]. For this test, the performance of the model is satisfactory when the p-value is greater than the significance level (0.05) and vice versa. The Chi-square and Hosmer and Lemeshow test have similar characteristics for testing the accuracy of a model. The multivariate models of the type of role and Socio-demographic characteristics (p = 0.944), the reasons for ASGM (p = 0.981) as well as the occurrence of injuries and risk factors (p = 0.554) the returned a p-value greater than the significance level (0.05) (see Table 2). As such, the results revealed that the model fitted the data accurately since the Chi-square significance level is greater than 0.05.

**Table 2:** Hosmer and Lemeshow good-for-fitness test.

Chi-square	Df	Level of Significance
**Type of role**		
1.717	6	0.944
**Reasons for ASGM**		
0.732	5	0.981
**Occurrence of injuries/accidents**		
6.836	8	0.554

### 2.4 Limitations of the study

The scope of the study was limited to Amansie central district in Ghana. However, the findings of the study can be generalized and/or replicated since Amansie central district presents similar characteristics of mining communities in Ghana and Africa. Again, the study only focused on ASGM and not large-scale mining. This is due to the cumbersome bureaucracies involved in attaining data in large-scale mines in Ghana.

## 3 Results

### 3.1 Socio-demographic characteristics of respondents

**Fig. 2:**
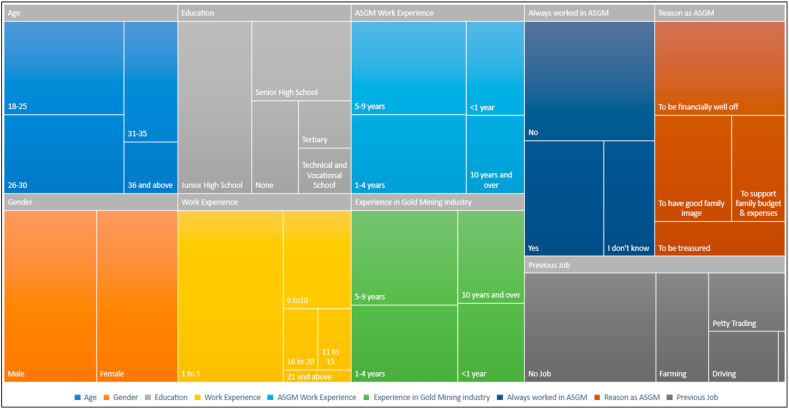
Treemap of socio-demographic characteristics of respondents.

Fig. 2 presents a treemap of the socio-demographic characteristics of respondents. Out of the 250 respondents, 94 respondents representing about 38% were between the ages of 18–25 years. Also, 79 of the respondents representing 32% were between the ages of 26–30 years. About 133 respondents representing 53% were males. The rest, on the other hand, were females. In terms of educational level, majority of the respondents (107) representing 42.8% had up to Junior high school. Approximately 21 (8.4%) of the respondents had tertiary education. However, about 36 (14.4%) of the respondents had no education. This reveals that the literacy level is fairly high in the study area. However, the number of tertiary levers who engage in ASGM is quite high (8.4%). Concerning work experience, majority of the respondents (60.8%) had 1–5 years of work experience (Fig. 2). This reveals that most of the respondents had at least some years of experience in ASGM. However, in terms of work experience, about 90 (36%) of the respondents had about 5–9 years of experience. This shows that most of the respondents have good experience in ASGM. This is also in consonance with the findings of the study concerning respondents’ overall experience in the gold mining industry. Similarly, most of the respondents had about 5–6 years of experience in the gold industry. For reasons why the respondents engage in ASGM, majority of the respondents (40%) revealed that they engage in ASGM to be financially well off (Fig. 2). However, most of the respondents (50.8%) claimed they have not worked in the ASGM. For the previous jobs of respondents, about 50.4% of the respondents representing the majority noted that they had no job (Fig. 2).

### 3.2 Socio-demographic characteristics of respondents and their influence on the assignment of roles in ASGM

**Fig. 3:**
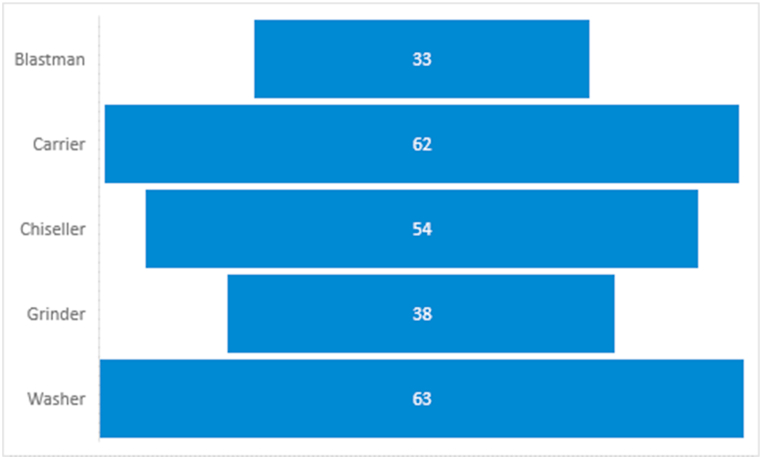
Funnel plot of the type of work in ASGM.

From Fig. 3, most of the respondents (63) representing about 25.2% of the total population were washers whereas 62 respondents representing about 24.8% were Carriers. This may be attributed to the number of females who engage in ASGM. Also, most newbies and older people in the ASGM are often given the role of washers or carriers. This is because these roles require no relevant experience to be discharged.

For this reason, the study tested the hypotheses of socio-demographic characteristics on the assignment of respondents’ roles using the Pearson Chi-square and likelihood ratio test as well as the binary regression model.

Table 3 reveals that blastman (96.7%), Chiseler (94%), and grinder (81.6%) roles are often given to the males in ASGM. However, roles such as Carrier and Washer roles are dominated by females (see Table 3) compared to the males. The association between gender and the type of role was statistically significant at 1% significance level (χ2 = 66.08, df = 4, p = 0.000; LR = 75.41, df = 4, p = 0.000). Therefore, we reject the null hypothesis and accept the alternative hypothesis that gender influences the assignment of roles in ASGM. Similarly, the multivariate model (Table 4) revealed that male miners had a higher likelihood of being assigned a type of role. This association was statistically significant at 5% level (p = 0.001) with AOR of 0.068, implying that gender influences the type of role assigned. This may be due to the energy-intensive nature of ASGM, site managers prefer employing men compared to women.

**Table 3:** Demographic characteristics versus type of roles.

Demographic Characteristics		Type of roles					Total	Test	Value	df	*P*-Value
		Blastman	Carrier	Chiseller	Grinder	Washer					
Gender											
Female	Frequency	1	30	3	7	46	87	Pearson Chi-Square (χ2)	66.078	4	0.000
	Percentage	3.3%	56.6%	6.0%	18.4%	58.2%	34.8%	Likelihood Ratio (LR)	75.413	4	0.000
Male	Frequency	29	23	47	31	33	163				
	Percentage	96.7%	43.4%	94.0%	81.6%	41.8%	65.2%				
Total	Frequency	30	53	50	38	79	250				
	Percentage	100.0%	100.0%	100.0%	100.0%	100.0%	100.0%				
**Age**											
18–25	Frequency	2	12	23	14	26	77	Pearson Chi-Square (χ2)	74.010	32	0.000
	Percentage	6.7%	22.6%	46.0%	36.8%	32.9%	30.8%	Likelihood Ratio (LR)	65.929	32	0.000
26–30	Frequency	10	12	14	16	27	79				
	Percentage	33.3%	22.6%	28.0%	42.1%	34.2%	31.6%				
31–35	Frequency	15	10	11	7	20	63				
	Percentage	50.0%	19.0%	22.0%	18.5%	25.3%	25.2%				
36 and above	Frequency	3	19	2	1	6	31				
	Percentage	10.0%	35.8%	4.0%	2.6%	7.6%	12.4%				
Total	Frequency	30	53	50	38	79	250				
	Percentage	100.0%	100.0%	100.0%	100.0%	100.0%	100.0%				
**Education**											
Junior High School	Frequency	14	15	20	19	26	94	Pearson Chi-Square (χ2)	21.939	16	0.145
	Percentage	46.7%	28.3%	40.0%	50.0%	32.9%	37.6%	Likelihood Ratio (LR)	23.567	16	0.099
None	Frequency	2	10	10	6	8	36				
	Percentage	6.7%	18.9%	20.0%	15.8%	10.1%	14.4%				
Senior High School	Frequency	4	10	14	9	24	61				
	Percentage	13.3%	18.9%	28.0%	23.7%	30.4%	24.4%				
Technical and Vocational School	Frequency	7	11	3	3	14	38				
	Percentage	23.3%	20.8%	6.0%	7.9%	17.7%	15.2%				
Tertiary	Frequency	3	7	3	1	7	21				
	Percentage	10.0%	13.2%	6.0%	2.6%	8.9%	8.4%				
Total	Frequency	30	53	50	38	79	250				
	Percentage	100.0%	100.0%	100.0%	100.0%	100.0%	100.0%				
**ASGM work Experience**											
<1 year	Frequency	2	19	13	15	25	72	Pearson Chi-Square (χ2)	29.211	12	0.004
	Percentage	6.7%	35.8%	26.0%	39.5%	31.6%	28.8%	Likelihood Ratio (LR)	31.008	12	0.002
1–4 years	Frequency	4	13	20	12	21	70				
	Percentage	13.3%	24.5%	40.0%	31.6%	26.6%	28.0%				
10 years and over	Frequency	8	4	7	6	11	36				
	Percentage	26.7%	7.5%	14.0%	15.8%	13.9%	14.4%				
5–9 years	Frequency	16	17	10	5	22	72				
	Percentage	53.3%	32.1%	20.0%	13.2%	27.8%	28.8%				
Total	Frequency	30	53	50	38	79	250				
	Percentage	100.0%	100.0%	100.0%	100.0%	100.0%	100.0%

**Table 4:** The association between socio-demographic features and the type of roles.

Socio-demographic characteristics	COR (95% CI)	AOR (95% CI)	Multivariate p-value
Gender			
**Male** = **1**	0.081(0.042–0.178)	0.068(0.032–0.14)	0.001
**Female** = **2**			
Age groups			
**18**–**35 years** = **1**	0.310(0.097–0.633)	0.217(0.078–0.60)	0.003
**35 years and above** = **2**			
Work Experience			
**1**–**10years** = **1**	0.283(0.161–0.473)	1.315(0.584–2.96)	0.011
**11 years and above** = **2**			
Educational level			
**None, primary, Junior high, Technical (Low)** = **1**	0.807(0.546–0.927)	0.531(0.281–1.00)	0.050
**Senior high, Tertiary (High)** = **2**			

In terms of age and the type of role, respondents between the ages of 31–35 years (50%) were given more of the blastman role out of the 30 respondents who were blastmen (see Table 3). Within the carriers, majority of the respondents (35.8%) were between the ages of 36 and above years. Table 3 also reveals that most of the respondents (46%) within chisellers were between the ages of 18–25 years. Within grinders and washers, most of the respondents (42.1%) and (34.2%) respectively were between the ages of 26–30 years. This shows that most of the roles that require more energy are given to the younger miners. The relationship between age and the type of role was statistically significant at a 1% significance level for both tests (χ2 = 74.01, df = 32, p = 0.000; LR = 65.93, df = 32, p = 0.000). Therefore, we reject the null hypothesis and accept the alternative hypothesis that age influences the assignment of roles in ASGM. Similarly, the multivariate model found that the youth in the age group between 18 and 35 years had a higher tendency of being assigned a type of role. This attained a statistically significant relationship with p < 0.05 and AOR of 0.217 (Table 4). This implies that age influences the type of role assigned to the youth.

For educational level and the type of role, most of the respondents (46.7%) within the blastman role had junior high school education. Similarly, majority of the respondents within the other type of roles such as Carrier (28.3%), Chiseler (40%), Grinder (50%) and Washer (32.9%) had up to Junior high school education (see Table 3). For senior high school levers, most of the respondents with this qualification were assigned washers and followed by a chiseller role. This is similar to the roles given to the tertiary education holders who were given carrier (13.2%) and washer (8.9%) roles. However, respondents with no education were given the carrier and chiseler role in the study area. However, the association between educational level and the type of role was not statistically significant at 5% based on the Pearson Chi-square and the Likelihood ratio test. Therefore, we fail to reject the null hypothesis that educational level has no influence on the assignment of roles in the ASGM and reject the alternative hypothesis based on the Pearson Chi-square (χ2 = 21.94, df = 16, p = 0.145; LR = 23.57, df = 16, p = 0.099). The multivariate model further revealed that miners with low education levels had a high tendency of being assigned a type of role. This was statistically significant (p = 0.050) with AOR of 0.531 (Table 4).

For the number of years of experience in ASGM and the type of role, most of the respondents (53.3%) within the blastmen had 5–9 years of experience (Table 3). This is followed by miners with 10 and above years of experience. This may be due to the skill requirement of blasting in ASGM. Because these skills are attained on the job, most of the blasting activities are done by skillful and more experienced miners. Moreover, most of the respondents (35.8%) had less than one year of work experience within the carrier role. This is followed by 32.1% of the respondents with 5–9 years of experience (Table 3). This may be due to the low skills requirement for the carrier role. Therefore, the newly recruited miners are given such roles as a startup. Also, due to the low energy requirement of the carrier role, some of the older miners choose to remain in this position for years. For the chiseller role, approximately 40% of the respondents had 1–4 years of experience (Table 3). This is followed by 26% of the respondents with less than one year of experience. The chiseller role requires fewer skills, however, more energy to be done. Therefore, respondents with fewer skills but energetic are likely to be given the chiseller role. For the grinder role, majority of the respondents (39.5%) had less than one year of experience (Table 3). This may be largely due to the energy requirement of the grinder role. As a result, most of the miners assigned to this role either switch to other roles or quit. The physical injuries, as well as the health repercussions, may be the major reason why most of them quit or move to other roles. The association between ASGM work experience and the type of role was statistically significant at 1% significance level (χ2 = 29.21, df = 12, p = 0.004; LR = 31.01, df = 12, p = 0.002) (Table 3). In terms of work experience, miners with 1–10 years of work experience dominated the type of role as revealed by the multivariate model. The association between work experience and type of roles was statistically significant (p = 0.011, AOR = 1.315) (see Table 4).

Table 4 shows the COR and AOR of socio-demographic features against the type of role. The results found a positive and statistically significant relationship between the socio-demographic characteristics (such as gender, age, work experience, and education level) and type of roles since p < 0.05. This signifies that socio-demographic characteristics influence the assignment of roles in the ASGM.

### 3.3 The influence of socio-demographic characteristics of respondents on the reasons for engaging in ASGM

The COR and AOR of socio-demographic features of respondents against the reasons for ASGM are presented in Table 5. The results reveal the associations between socio-demographic features and the reason for ASGM. However, these associations were not statistically significant at a 5% significance level. This implies that socio-demographic features used in this study do not influence the reason for ASGM.

**Table 5:** Reason for engaging.

Socio-demographic characteristics	COR (95% CI)	AOR (95% CI)	Multivariate p-value
**Gender**			
Male = 1	1.209(0.717–2.039)	1.219(0.71–2.066)	0.462
Female = 2			
**Age groups**			
18–35 years = 1	1.158(0.545–2.458)	1.145(0.535–2.452)	0.727
35 years and above = 2			
**Work Experience**			
1–10years = 1	1.587(0.815–3.091)	1.595(0.817–3.114)	0.171
11 years and above = 2			
**Educational level**			
None, primary, Junior high, Technical (Low) = 1	1.152(0.678–1.957)	1.151(0.674–1.965)	0.606
Senior high, Tertiary (High) = 2			

Table 5 showed that the males had a higher likelihood of engaging in ASGM. This may be due to the energy requirements of ASGM. This makes men a preferable choice compared to women. Miners in the age group between 18 and 35 years had a higher tendency of engaging in ASGM. Again, younger people with adequate strength to undertake the rigorous activities of ASGM may be the main reason. Moreover, people with 1–10 years of working experience had a higher chance of engaging in ASGM. This may be due to the high rate of unemployment amongst the youth in developing countries such as Ghana. Finally, people with low education levels had a higher tendency of engaging in ASGM. This may also be related to unemployment issues prevailing currently in developing countries such as Ghana. However, the association between socio-demographics and reasons for ASGM was statistically insignificant at a 5% level (see Table 5).

### 3.4 Occupational health and safety hazards associated with ASGM

The occupational health and safety hazards associated with ASGM were assessed. Approximately, 60.8% of the respondents representing the majority indicated that they are aware of the occupational health and safety hazards associated with their job (Table 6). Most of the respondents further indicated that they know about the fall of rocks as the most occurring occupational health and safety hazard in ASGM. This was statistically significant at 1% significance level (χ2 = 241.96, df = 5, p = 0.000; LR = 316.62, df = 5, p = 0.000) (Table 6).

**Table 6:** Respondents’ knowledge about occupational health and safety hazards in ASGM.

Knowledge about occupational health hazards in ASGM		Kinds of Hazards						Total
			malaria and dengue fever	psychophysical impairment	Rock fall	shoulder disorder	Skin splashes with cyanide	
No	Frequency	96	0	0	1	0	1	98
	Percentage	100.0%	0.0%	0.0%	1.1%	0.0%	7.1%	39.2%
Yes	Frequency	0	18	22	89	10	13	152
	Percentage	0.0%	100.0%	100.0%	98.9%	100.0%	92.9%	60.8%
Total	Frequency	96	18	22	90	10	14	250
	Percentage	100.0%	100.0%	100.0%	100.0%	100.0%	100.0%	100.0%
**Test**	**Value**	**df**	***P*-Value**					
Pearson Chi-Square (χ2)	241.955	5	0.000					
Likelihood Ratio (LR)	316.624	5	0.000					

Regarding the awareness of the use of PPE, about 49.6% of the respondents revealed that they know about PPE (Table 7). Also, in defining what PPE is used for, most of the respondents who claimed “Yes” revealed that PPE is used for the protection of the body from occupational health and safety hazards associated with ASGM. However, the rest of the respondents noted that PPE is used for sampling, and washing and some did not even know the use of PPE. This shows that, despite the advancement in PPE technology, some miners in the ASGM lack knowledge about its usage. This may be the main cause of the low patronage of PPE in the ASGM. This was statistically significant at 1% significance level (χ2 = 230.76, df = 12, p = 0.000; LR = 304.09, df = 12, p = 0.000) (Table 7).

**Table 7:** Respondents' Awareness and usage of PPE.

Awareness of PPE		Usage of PPE					Total
			Don’t know	Protection	Sampling	Washing	
	Frequency	1	0	0	0	0	1
	Percentage	0.8%	0.0%	0.0%	0.0%	0.0%	0.4%
Can't remember	Frequency	50	0	0	0	0	50
	Percentage	38.2%	0.0%	0.0%	0.0%	0.0%	20.0%
No	Frequency	75	0	0	0	0	75
	Percentage	57.3%	0.0%	0.0%	0.0%	0.0%	30.0%
Yes	Frequency	5	4	94	12	9	124
	Percentage	3.8%	100.0%	100.0%	100.0%	100.0%	49.6%
Total	Frequency	131	4	94	12	9	250
	Percentage	100.0%	100.0%	100.0%	100.0%	100.0%	100.0%
**Test**	**Value**	**df**	***P*-Value**				
Pearson Chi-Square (χ2)	230.762	12	0.000				
Likelihood Ratio (LR)	304.093	12	0.000				

On the usage of PPE by the miners, most of the respondents (60%) representing 150 respondents revealed that they do not use PPE during their operations (Table 8). Out of this, about 77 of the respondents revealed that they mostly record injuries from being struck by rocks during their operations (Table 8). This was followed by falls, slips, and trips from height (see Table 8). The relationship between the use of PPE and the kind of injuries that occur was statistically significant at a 5% significance level (χ2 = 19.40, df = 8, p = 0.013; LR = 17.10, df = 8, p = 0.029) (Table 8).

**Table 8:** Respondents present use of PPE and the most occurring kinds of injuries/accidents in ASGM.

Present use of PPE		Most occurring kinds of injuries/accidents in ASGM					Total
		being hit by a moving object	being struck a by rock	being struck by a metallic object	fall/slip/trip from height	vehicle rollover	
No	Frequency	12	77	22	33	6	150
	Percentage	75.0%	58.3%	73.3%	63.4%	30.0%	60.0%
Yes	Frequency	4	55	8	19	14	100
	Percentage	25.0%	41.7%	26.7%	36.5%	70.0%	40.0%
Total	Frequency	16	132	30	52	20	250
	Percentage	100.0%	100.0%	100.0%	100.0%	100.0%	100.0%
**Test**	**Value**	**df**	***P*-Value**				
Pearson Chi-Square (χ2)	19.401	8	0.013				
Likelihood Ratio (LR)	17.100	8	0.029				

Table 9 presents the COR and AOR of risk factors against the occurrence of injuries/accidents in ASGM. The results revealed that there is a positive association between the occurrence of injuries/accidents in ASGM and several risk factors such as the socio-demographic characteristics of respondents, type of role, reasons for ASGM, awareness of occupational health hazards, awareness of PPE, usage of PPE, arrest for not using PPE, cost of PPE and frequency of buying PPE since (p < 0.05) in the multivariate model. This implies that significant risk factors have an influence on the occurrence of injuries/accidents. However, other risk factors were not statistically significant (see Table 9). Table 9 comprises statistically significant risk factors that influence the occurrence of injuries/accidents. All statistically insignificant risk factors were manually removed.

**Table 9:** Risk factors for the Occurrence of injuries/accident.

Risk factors	COR (95% CI)	AOR (95% CI)	Multivariate p-value
Gender			
**Male** = **1**	0.076(0.037–0.154)	0.035(0.013–0.092)	0.000
**Female** = **2**^**RC**^			
Age groups			
**18**–**35 years** = **1**	0.229(0.091–0.581)	0.201(0.066–0.612)	0.005
**36 years and above** = **2**^**RC**^			
Work Experience			
**1**–**10years** = **1**	0.283(0.061–0.493)	0.241(0.019–0.407)	0.004
**11 years and above** = **2**^**RC**^			
Educational level			
**None, primary, Junior high, Technical (Low)** = **1**^**RC**^	0.708(0.416–1.207)	0.269(0.118–0.615)	0.002
**Senior high, Tertiary (High)** = **2**^**RC**^			
Type of role			
**Washer, carrier** = **1**	0.885(0.315–0.921)	0.470 (0.569–0.930)	0.037
Blastman, chiseller, grinder = 2^RC^			
Reasons for ASGM			
**To be financially well off, To be treasured** = **1**	0.286(0.339–0.954)	0.245(0.213–0.992)	0.011
**To have a good family image, To support family budget & expenses** = **2**^**RC**^			
Awareness of occupational health hazards in ASGM			
**Yes** = **1**^**RC**^			
**No** = **2**	0.172(0.045–0.162)	0.146(0.023–0.112)	0.001
Awareness of PPE			
**Yes** = **1**^**RC**^			
**No, Can't remember** = **2**	0.812(0 .493–0.936)	0.796(0.391–0.619)	0.029
Usage of PPE			
**Yes** = **1**^**RC**^			
**No** = **2**	0.618(0.327–0.875)	0.575(0.214–0.844)	0.046
Arrest for not using PPE			
**No** = **1**	0.434(0 .216–0.735)	0.666(0.376–0.878)	0.031
**Nobody checks this** = **2**^**RC**^			
Cost of PPE			
**Gh100**–**200, 250–300, 350–400** = **1**	0.257(0.185–0.488)	0.504(0.209–0.917)	0.028
**450**–**500, 650–700** = **2**^**RC**^			
Frequency of buying PPE			
**Yearly, As and when it gets worn out** = **1**	0.082(0.029–0.177)	0.055(0.018–0.097)	0.008
**Monthly** = **2**^**RC**^			

### 3.5 Reasons for the refusal to use PPE in ASGM

The respondents’ reasons for not using PPE in the operations of ASGM are presented in Table 10. Despite the advantages of using PPE in ASGM, Table 10 reveals that most of the respondents do not use PPE because of the high cost associated with the purchase of PPE in Ghana. A mean response value of 1.92 was attained (Table 10).

**Table 10:** Respondents’ reasons for not using PPE.

	N	Mean	Std. Deviation	Legend
Reason for not using PPE	250	1.92	1.04	1 = Uncomfortable to use PPE 2 = High cost 3 = Waste of time 4 = Unavailable
Cost of PPE	250	2.40	1.78	1 = 250 - 300 2 = 350–400 3 = 450–500 4 = 550 - 600
Availability of PPE on local market	250	1.41	0.49	1 = Yes 2 = no
Colleagues arrested for not using PPE	249	1.71	0.45	1 = Yes 2 = No

The respondents indicated that a PPE may cost between 350 and 400 Ghana cedis with 2.40 attained as a mean response value (Table 10). This shows that the cost of getting PPE is quite high in the context of Ghana. Also, the respondents indicated that none of their colleagues have been arrested for not using PPE. A mean response value of 1.71 was attained (Table 10). This may be one of the major causes of why most miners do not use PPE in their operational activities. However, most of the respondents revealed that PPE is readily available in their local markets with a means response value of 1.41.

## 4 Discussion

### 4.1 Socio-demographic characteristics of respondents

Generally, the results reveal that most of the respondents fall within the working age (18–35 years) and were males. This is in line with the findings of Mensah et al. [10], that the ASGM industry has a significant gender gap because men predominate. This may be due to the manpower required in the ASGM. ASGM requires youthful miners due to the intensity of energy required and the enthusiastic youthful population in local areas is strategically fit [[30], [31], [32], [33]]. This is in line with the findings of Hentschel and Priester [20] that a chunk of the active population engaged in ASGM emanates from developing countries like Ghana. It has been established that ASGM offers jobs to 13 million people [15,34] and serves as a source of income for 80–100 million people globally [15,34]. With a chunk of ASGM operators emanating from developing countries like Ghana [20,34]. However, the findings of the study regarding age contradict that of Boafo et al. [35] that in Ghana, children account for the majority of ASGM workers.

Moreover, literacy level was found to be fairly high since the number of tertiary levers who engage in ASGM was revealed to be quite high. This may be attributed to the high unemployment rate in Ghana where many graduates find it difficult to find jobs as confirmed by the findings of Arthur-Holmes and Abrefa [4]. Another study by Arthur-Holmes et al. [36], found that a growing number of graduates in Ghana have entered the ASM industry as a result of the rising graduate unemployment rate. Conversely, this contradicts the findings of previous studies [10,33,37] that ASGM mostly employs people who have less specialty, often found in rural areas with poor educational levels. Baddianaah et al., [38], found that when compared to individuals with no formal education, people with a first degree are 0.24 times less likely to participate in ASGM undertakings. Most of the respondents selected for the study had at least some years of experience in ASGM. Therefore, can offer relevant information needed for the study.

### 4.2 Evaluation of the influence of socio-demographic characteristics on role assignment in ASGM

Majority of the respondents were washers and carriers. These roles are usually assigned to females, newbies, and older people who engage in ASGM. This is because these roles require no relevant skills to be employed. This is in line with the hypothesis test where roles such as washers and carriers were dominated by females, older and less experienced people. This may be due to the energy requirements of certain types of work in the ASGM which may not be appropriate for certain gender (especially females), age-groups, and levels of experience. As a result, the findings further show that, due to the low energy requirement of the washer and carrier role, some of the older miners choose to remain in this position for years. However, the low skills requirement for the washer and carrier role may be a factor. Therefore, the newly recruited miners were found to be given such roles as a startup. The findings of the study align with that of [[39], [40], [41]] that because new miners with less work experience are engaged to carry and load ore into washing plants, women largely remain contract employees in ASGM undertakings. Amoako et al. [42] found that ASGM is more dependent on the available manpower than capital compared to large-scale mining. Therefore, the younger miners in terms of age are used for energy-intensive roles [10] such as chiseling, grinding, etc. Compared to the older ones.

Most of the respondents had Junior and Senior high school education. This may be due to the Junior and Senior high school students who might have completed their education and looking for a temporal job to do. As a result, due to their inexperience on the job, they are given roles that do not require any relevant skills. However, the tertiary respondents (graduates) were given lighter roles such as washers and carriers. Due to the lack of availability of jobs for graduates in Ghana, some of them who have no choice land themselves in the ASGM as temporary jobs to survive [36]. Existing studies [43,44] noted that the booming of ASGM in the face of a high unemployment rate discourages the youth to pursue formal education. This may be attributed to the fact that ASGM does not require any special skills to be employed. ASGM is noted to require no special skills nor education to be employed and as a result, employs people especially those in remote areas [37].

### 4.3 Respondents’ reasons for engaging in ASGM

The study reveals that socio-demographic features influenced the reasons for ASGM. This is in line with the findings of previous studies [43,45,46] that ASGM provides direct income and far more tangible benefits than industrial mining. This provides immediate income needed for rural people struggling to escape poverty and hence improves the standard of living and health by enabling them to access good health care for themselves. While some miners aspired to be wealthy, the majority were obliged to work in the mines because they had no other means to support their families [47]. Most Artisanal miners, according to Ref. [48], labor to provide for their family's needs. ASGM contributes to the creation of a significant number of indirect jobs in other sectors of the economy in addition to direct employment possibilities [7,40,[49], [50], [51]].

The findings of Hilson et al., and Jennings [15,34] revealed that about 80–100 million people rely on ASGM for a living either directly or indirectly. In some rural communities in Ghana, ASGM is the livelihood of the people as opposed to agriculture (e.g. Refs. [26,[52], [53], [54], [55]]). It provides a social "safety net" for the unemployed and those with no other options, as well as subsistence farmers hit by drought [56]. Moreover, ASGM is frequently regarded as the sole means of reducing poverty in many locations where there are inadequate alternative economic options for the populace.

### 4.4 ASGM and its associated occupational health and safety hazards

The results showed that most of the respondents are aware of the occupational health and safety hazards associated with ASGM. Despite majority of the respondents knowing what PPE is, majority of the respondents do not use PPE whereas some lacked knowledge and education about the use of PPE in the operational activities of ASGM. However, the fall of rocks followed by falls, slips, and trips from height respectively were identified as the most occurring hazards. This shows that the respondents who refuse to use PPE are the most vulnerable to hazards such as rockfalls, slips, falls, and trips from height in the ASGM. This is in line with the study by Marriot [57] that rockfall from above is the leading factor responsible for a high rate of accidents and injuries. However, Long et al. [58], revealed that major factors such as wrong use of explosives, collapsing of mine tunnel/structure, insufficient workspace, poor ventilation, trips, and fall, etc. were major hazards resulting in mine accidents with explosive blasts being the most challenging of all. Hilson and Mcquilken [59] also found that ASGM activities from extraction to processing require the use of rudimentary techniques exposing miners to all forms of hazards. However, there is a lack of safety requirements in the identification of hazards, use of mining instruments, and the execution of health and safety standards [4,15].

Table 9 revealed that the male miners had a higher risk of recording injuries/accidents (p = 0.000, AOR = 0.035). Different studies conducted in Zimbabwe [60] and Ethiopia [61] found that male miners are 15.3 and 2.54 times respectively more exposed to severe injuries compared to female miners. This is in line with the findings of Ajith and Ghosh [62] that male miners are noted for riskier duties such as using rudimentary tools to dig into unlit and unventilated pits compared to the female miners who work in an open space with fewer energy-intensive roles. Similarly, other studies suggest that ASGM is entirely a male-dominated undertaking, with females mostly working in menial positions at ASGM locations [63,64].

Moreover, the results showed that respondents between 18 and 35 years had a higher risk of recording injuries/accidents compared to those older than 36 years and above. This is statistically significant at 5% (p = 0.005) with an AOR of 0.201. The results agree with the findings of [62,65,66] that younger miners are more susceptible to the occurrence of injuries/accidents. This may be a function of the work experience, education level and type of role of miners. The results show that miners with less work experience between 1 and 10years (p = 0.004, AOR = 0.241), a low education level (None, primary, Junior high, Technical) (p = 0.002, AOR = 0.269) and assigned to a role that requires less skills such as Washers and carriers (p = 0.037, AOR = 0.470) are likely to record more injuries/accidents. Ajith and Ghosh [62] found that the younger miners with work experience fail to identify and manage hazards and have careless behavior. However, previous studies [62,65] found that the younger miners suffer less severe injuries than the older miners. This may be attributed to the less dangerous roles such as washers, carriers, etc. given to the younger miners.

For the reasons for ASGM as a risk factor, miners who engaged in ASGM to be financially well off had a higher risk (p = 0.011, AOR = 0.245) of recording injuries/accidents. These are mostly inexperienced young miners with fewer family responsibilities who are so desperate to be financially well off. As a result, they engage in riskier prospects to acquire more wealth at the expense of their health and safety. Less experienced miners are noted to have less knowledge about their new environment and its related hazards [62].

Table 9 further reveals that miners with less awareness of occupational health hazards (p = 0.001, AOR = 0.146) and PPE (p = 0.029, AOR = 0.796) in ASGM had a higher risk of recording injuries/accidents. The studies of Calys-Tagoe et al. [67] and Chimamise et al. [60] revealed that the rudimentary tools, availability of hazards without appropriate control techniques, lack of safety training, lack of policies and social hazards as a result of poor perception of management and supervision leads to severe and frequent injuries [68].

Concerning the usage of PPE, miners who do not use PPE had a higher risk of recording injuries/accidents. The relationship was statistically significant at 5% level (p = 0.046, AOR = 0.575) (Table 9). This may be attributed to monitoring agencies not arresting miners who refuse to use PPE. As a result, the respondents who were not arrested for not using PPE had a higher risk (p = 0.031, AOR = 0.666) of recording injuries/accidents. Miners who may not be arrested end up mining without PPE which exposes them to frequent and severe injuries. Aside from this, failure to arrest defaulters may be a cause as most respondents indicated that none of their colleagues have been arrested for not using PPE. Due to the weak institutional structure of agencies responsible for the arrest and prosecution of recalcitrant, the enforcement of the use of PPE has been unrestricted. If laws and policies on the use of PPE were strictly enforced, concession owners, as well as site managers, would be forced to equip miners with PPE and take into consideration all health and safety measures before and during their ASGM operations.

The MinCom and EPA are Ghana's two government agencies in charge of policing mining operations to guarantee a sustainable environment. Therefore, in an attempt to find out the actual roles played by the MinCom in the safety and health of ASGM revealed that the MinCom Act, 1993 (Act 450) and the Mining and Minerals Act, 703 of 2006 (Anon 2016b as cited by Ref. [69] are both accountable for overseeing and monitoring Ghana's mineral wealth. Besides that, under the Minerals and Mining (Health, Safety and Technical Regulations) LI 2182, (2012) Regulation 543 (1) A mine operator or owner of a small-scale mining license must supply PPE per each mine worker, and the operator or owner is liable for such use. (2) An individual shall not enter or stay in a safety helmet area of a mine, nor cause or induce another person to enter or stay there, but if that person or that other person is wearing a hard helmet in satisfactory condition and of a kind certified by the Chief Inspector. (3) Each worker who operates in a mine or accesses a mine must put on a pair of protective toe boots or shoes that have been certified by the Chief Inspector. (4) In the course of duty, anyone working in a sector of a mine where there may be a risk of eye harm must wear goggles. (5) A mine operator or owner of a small-scale mining license must guarantee that everyone employed in the mine wears or uses PPE, clothing, or gadget that the Chief Inspector of Mines has certified for each danger in the mine [70].

In terms of the cost of PPE as a risk factor, miners who bought less expensive PPE had a higher risk (p = 0.028, AOR = 0.504) of recording injuries/accidents (Table 9). This may be attributed to cheap PPE which may not be adequate to prevent some minor and severe injuries/accidents. Again, miners who bought PPE yearly/as and when worn out had a higher risk of recording injuries/accidents. This may be attributed to monitoring agencies’ failure to monitor the quality of PPE used in the ASGM leading to the use of cheap PPE which may be worn out after short usage rendering miners susceptible to the occurrence of injuries/accidents.

The findings of the study fall in line with previous findings [60,[71], [72], [73]] that PPE such as hardhats or helmets, gloves, safety spectacles, work boots, dust masks amongst others as easily available alternatives for reducing occupational related hazards, however, largely relegated and not essential, particularly in ASGM undertakings. As a result, miners may not want to spend considerable amounts on purchasing PPE. Mensah et al. [10] revealed that PPE safety training is not part of the factors that influence the uptake of occupational health and safety practices in ASGM. This may be due to the absence of safety pieces of training before and during ASGM operational activities [10]. The general perception that ASGM requires no training and that a person is trained while on the job may also be a cause.

### 4.5 Reasons for not using PPE

Majority of the respondents do not use PPE due to the high cost associated with their purchase. The cost of a PPE is estimated between 350 and 400 cedis. Most concession owners or site managers fail to purchase PPE such as safety helmets, boots, overall suits, gloves, goggles, respirators, etc., and organize safety training for their workers because of the cost involved. Therefore, miners are left at the mercy of all manners of injuries. As a result, miners are mostly found to leverage on rudimentary means of protecting themselves from accidents and injuries.

The study further noted that the MinCom supervises and monitors ASGM operations to enforce safety laws, as evidenced by an interview with a desk officer. It was also noted that the MinCom offers miners safety awareness and support. However, according to Amoako et al., [42], the Minerals and Mining Act 703 was designed to encourage efficient mining standards and allow strong global recognition of the mining sector of Ghana, but considerably, has been unsuccessful. The regulation, in contrast, does not always seem to be assisting the country's development of long-term and equitable ecological sustainability [35].

It is noted that Ghana just like major African countries are grappling in the area of OHS practices as governments and corporations have made insignificant efforts. The study recommends that periodic safety training be organized by accountable regulators for ASGM miners to help foster the uptake of OHS practices. Also, the provision of adequate resources to regulatory authorities to monitor and supervise ASGM activities effectively and efficiently.

## 5 Conclusion

The study assessed the socio-demographic effects on role assignment and associated Occupational health and safety issues in artisanal and small-scale gold mining in Amansie Central District, Ghana. The study sampled 250 respondents using the simple random sampling technique. A descriptive research design together with a field survey focused on socio-demographic characteristics of respondents and OHS issues were employed. The binary logistic regression model as well as the Chi-square and likelihood ratio test were used to examine the association between the socio-demographic characteristics and the occurrence of injuries/accidents. The socio-demographic characteristics of ASGM workers, such as age, gender, work experience, and education, have been identified as important factors influencing the type of role assigned to individuals in ASGM and the associated OHS risks. In terms of the occurrence of injuries/accidents, all the socio-demographic factors were found to be risk factors for the occurrence of injuries/accidents. For the other factors, miners who engaged in ASGM had a higher risk of recording injuries/accidents. Also, miners with less awareness of occupational health hazards and PPE as well as not using PPE had a higher risk of recording injuries/accidents. Moreover, miners who were not arrested for not using PPE, and bought less expensive PPE yearly or as and when needed had a higher risk of recording injuries/accidents. The findings of the study will provide insight into how socio-demographic characteristics shape the assignment of roles and its associated OHS issues in ASGM, especially in Ghana. The results will inform strategies to promote OHS in ASGM, such as the development of targeted interventions to reduce occupational risks. The findings of this study will also be relevant to other contexts where ASGM is practiced. The results will help to identify the socio-demographic characteristics that shape the assignment of roles and its associated OHS issues in ASGM and inform strategies to address these issues.

### 6 Recommendations

1. The government, Regulatory bodies (MinCom & EPA), and stakeholders concerned in local communities (e.g., Municipal and Districts Assemblies (MDAs)) increase the number of training and education programs related to OHS risks in ASGM, targeting individuals of various ages and genders.
2. Establish a better understanding between employers and workers of the legal rights and responsibilities related to OHS in ASGM operations.
3. Develop specific policies on OHS risks in ASGM operations in Ghana, focusing on workers from different socio-demographic groups.
4. Implement measures to improve the work environment, such as providing protective equipment and creating safe working conditions.
5. Invest in research to understand better the complex interactions between socio-demographic factors, decision-making processes, and OHS risks in ASGM.
6. The government should also provide incentives for miners to access PPE at an affordable cost.

### Author contribution statement

Peter Donkor: Conceived and designed the experiments; Analyzed and interpreted the data; Wrote the paper.

Ebenezer Kwadwo Siabi: Performed the experiments; Contributed reagents, materials, analysis tools or data; Wrote the paper.

Kwasi Frimpong: Contributed reagents, materials, analysis tools or data.

Samuel Kofi Mensah: Performed the experiments.

Sarah Elikplim Siabi; Christopher Vuu: Analyzed and interpreted the data.

## Funding statement

This research did not receive any specific grant from funding agencies in the public, commercial, or not-for-profit sectors.

## Data availability statement

Data will be made available on request.

## Declaration of interest's statement

The authors declare no conflict of interest.

## References

[1] Hilson G., Hu Y. (2022). Changing priorities , shifting narratives : remapping rural livelihoods in Africa ’ s artisanal and small-scale mining sector. J. Rural Stud..

[2] Hilson G., Bartels E., Hu Y. (2022). Brick by brick , block by block : building a sustainable formalization strategy for small-scale gold mining in Ghana. Environ. Sci. Pol..

[3] Koomson-yalley E., Richard J., Owusu K. (2022). ‘ We are mine workers ’ : feminists ’ political economy in artisanal and small scale gold mining in the Talensi District , Ghana. J. Rural Stud..

[4] Arthur-holmes F., Arthur-holmes F., Abrefa K. (2022). Safety concerns and occupational health hazards of women in artisanal and small-scale mining in Ghana the Extractive Industries and Society Safety concerns and occupational health hazards of women in artisanal and small-scale mining in Ghana. Extr. Ind. Soc..

[5] Lydia O., Godwin A., Isaac L. (2022). The Extractive Industries and Society ‘ We have done nothing wrong ’ : youth miners ’ perceptions of the environmental consequences of artisanal and small-scale mining (ASM) in Ghana. Extr. Ind. Soc..

[6] WHO (2016).

[7] Abdulai A.-G. (2017). The galamsey menace in Ghana: a political problem requiring political solutions. Poli. Br.

[8] MyjoyOnline (2015). https://www.modernghana.com/amp/news/608372/we-need-a-deputy-minister-small-scale-miners.html.

[9] Siabi E.K., Mensah S.K., Donkor P., Kurantin N., Frimpong K., Siabi S.E., Etten E. Van, Vuu C. (2022). Assessing the knowledge and practices of occupational safety and health in the artisanal and small-scale gold mining sector of Ghana: A case of obuasi. Heliyon.

[10] Mensah K.S., Siabi K.E., Donkor P., Kurantin N. (2022). Assessing the safety and health practices in the artisanal and small-scale gold mining sector of Ghana : a case of Ntotroso. Environ. Challenges.

[11] Singo J., Moyo D., Isunju J.B., Reilly S.B. (2022). Health and safety risk mitigation among artisanal and small-scale gold miners in Zimbabwe. Int. J. Environ. Res. Publ. Health.

[12] Stemn E., Amoh P.O., Joe-Asare T. (2021). Analysis of artisanal and small-scale gold mining accidents and fatalities in Ghana. Resour. Pol..

[13] Ofosu G., Torbor M., Sarpong D. (2022). Gender and artisanal and small-scale mining : exploring women ’ s livelihood and occupational roles in formalised settings. J. Rural Stud..

[14] Owusu O., Joseph K., Kobina A. (2019). ‘ Small in size , but big in impact ’ : socio-environmental reforms for sustainable artisanal and small-scale mining. J. Sustain. Min..

[15] Hilson G., Hilson A., Maconachie R., Mcquilken J., Goumandakoye H. (2017). Geoforum Artisanal and small-scale mining (ASM) in sub-Saharan Africa : Re- conceptualizing formalization and ‘ illegal ’ activity. Geoforum.

[16] Hilson G., Hilson A., Maconachie R., McQuilken J., Goumandakoye H. (2017). Artisanal and small-scale mining (ASM) in sub-saharan Africa: Re- conceptualizing formalization and ‘illegal’ activity gavin. Geoforum.

[17] Bansah K.J. (2019). From diurnal to nocturnal : surviving in a chaotic artisanal and small-scale mining sector. Resour. Pol..

[18] Kinyondo A., Huggins C. (2021). State-led efforts to reduce environmental impacts of artisanal and small-scale mining in Tanzania: implications for fulfilment of the sustainable development goals. Environ. Sci. Pol..

[19] Hirons M. (2020). How the Sustainable Development Goals risk undermining efforts to address environmental and social issues in the small-scale mining sector. Environ. Sci. Pol..

[20] Hentschel T., Priester M. (2002). Global report on artisanal artisanal & small-scale mining thomas. Int. Inst. Environ. Dev..

[21] Danielsen K., Hinton J. (2020). Canadian Journal of African Studies/Revue canadienne A social relations of gender analysis of artisanal and small-scale mining in Africa ’ s Great Lakes Region A social relations of gender analysis of artisanal and small-scale. Can. J. Afr. Stud..

[22] MoFA Amansie Central. https://mofa.gov.gh/site/sports/district-directorates/ashanti-region/147-amansie-central.

[23] Amansie central district Assembly (2013).

[24] Wireko-Gyebi R.S. (2020). Perception of small-scale miners on interventions to eradicate illegal small-scale mining in Ghana. Sage Open.

[25] GSS (2021).

[26] Agariga F., Abugre S., Siabi E.K., Appiah M. (2021). Mining impact on livelihoods of farmers of asutifi North district, Ghana. Environ. Manag. Sustain. Dev..

[27] Likert R. (1932). A technique for the measurement of attitudes. Arch. Psychology.

[28] Hosmer D.W. (2000). Assessing the fit of the model. Appl. Logist. Regres..

[29] Ajith M.M., Ghosh A.K., Jansz J. (2021). A mixed-method investigations of work , government and social factors associated with severe injuries in artisanal and small-scale mining (ASM) operations. Saf. Sci..

[30] Hilson G. (2016). *Artisanal and small-scale mining and agriculture: Exploring their links in rural sub-Saharan Africa*.

[31] Hilson G., Maconachie R. (2020). Land use policy for the environment : an assessment of recent military intervention in informal gold mining communities in Ghana. Land Use Pol..

[32] Hilson G., Maconachie R. (2020). Entrepreneurship and innovation in Africa ’ s artisanal and small-scale mining sector : developments and trajectories. J. Rural Stud..

[33] (2015). Formalization of Small-Scale Mining in Ghana Gavin. *International Growth Center*, *April*.

[34] Jennings N. (1999).

[35] Boafo J., Paalo S.A., Dotsey S. (2019). Illicit Chinese small-scale mining in Ghana: beyond institutional weakness?. Sustainability.

[36] Arthur-holmes F., Abrefa K., Vazquez-brust D.A. (2022). Graduate unemployment , artisanal and small-scale mining , and rural transformation in Ghana : what does the ‘ educated ’ youth involvement offer. J. Rural Stud..

[37] Arthur F., Agyemang-Duah W., Gyasi R.M., Yeboah J.Y., Otieku E. (2016). Nexus between artisanal and small-scale gold mining and livelihood in prestea mining region, Ghana. Geogr. J..

[38] Baddianaah I., Nuoleyeng B., Adongo R. (2022). Heliyon Socio-demographic factors affecting artisanal and small-scale mining (galamsey) operations in Ghana. Heliyon.

[39] James McQuilken, Hilson Gavin (2016). *Evidence to inform an ‘action dialogue’.*.

[40] Bansah K.J., Yalley A.B., Dumakor-Dupey N. (2016). The hazardous nature of small scale underground mining in Ghana. J. Sust. Mining.

[41] Susapu B., Crispin G. (2002).

[42] Amoako C., Adarkwa K.K., Koranteng K.A. (2021). The politics of artisanal small-scale gold mining (ASM) in the Akyem Abuakwa Traditional Area of Ghana. J. Contemp. African Stud..

[43] Bazillier R., Girard V. (2020). The gold digger and the machine. Evidence on the distributive effect of the artisanal and industrial gold rushes in Burkina Faso. J. Dev. Econ..

[44] Ahlerup P., Baskaran T., Bigsten A. (2019). Gold mining and education : a long-run resource curse in Africa ? Gold mining and education : a long-run resource curse in Africa. J. Dev. Stud..

[45] Pokorny B., von Lübke C., Dayamba S.D., Dickow H. (2019). All the gold for nothing? Impacts of mining on rural livelihoods in Northern Burkina Faso. World Dev..

[46] Ingram V., Tieguhong J.C., Schure J., Nkamgnia E., Tadjuidje M.H. (2011). Where artisanal mines and forest meet: socio-economic and environmental impacts in the Congo Basin. Nat. Resour. Forum.

[47] Heemskerk M. (2011). Livelihood decision making and environmental degradation : small-scale gold mining in the Suriname amazon. Soc. Nat. Resour. An Int. J..

[48] Veiga M.M., Hinton J.J. (2002). Abandoned artisanal gold mines in the Brazilian Amazon : a legacy of mercury pollution. Nat. Resour. Forum.

[49] Amoah N., Stemn E. (2018). Siting a centralised processing centre for artisanal and small-scale mining – a spatial multi-criteria approach. J. Sustain. Min..

[50] Bansah K.J., Dumakor-dupey N.K., Kansake B.A., Assan E., Bekui P. (2018). Socioeconomic and environmental assessment of informal artisanal and small-scale mining in Ghana. J. Clean. Prod. J..

[51] Levin E. (2004). https://www.worldbank.org/en/topic/extractiveindustries/brief/artisanal-and-small-scale-mining.

[52] Kwai B., Hilson G. (2010). Livelihood diversification and the expansion of artisanal mining in rural Drivers and policy implications. Outlook Agric..

[53] Antwi-Boateng O., Akudugu M.A. (2020). Golden migrants : the rise and impact of illegal Chinese small-scale mining in Ghana. Polit. Pol..

[54] Osei L. (2017). https://ir.lib.uwo.ca/etd/5106.

[55] Agariga F., Abugre S., Appiah M. (2021). Spatio-temporal changes in land use and forest cover in the asutifi North district of ahafo region of Ghana, (1986–2020). Environ. Challenges.

[56] Bugnosen E., Twigg J., Scott A. (2000). Small-scale mining legislation and regulatory frameworks. Ind. Environ..

[57] Marriott A. (2008).

[58] Long R.N., Sun K., Neitzel R.L. (2015). Injury risk factors in a small-scale gold mining community in Ghana ’ s upper east region. Int. J. Environ. Res. Publ. Health.

[59] Hilson G., Mcquilken J. (2014). The Extractive Industries and Society Four decades of support for artisanal and small-scale mining in sub-Saharan Africa : a critical review. Biochem. Pharmacol..

[60] Chimamise C., Gombe N.T., Tshimanga M., Chadambuka A., Shambira G., Chimusoro A. (2013). Factors associated with severe occupational injuries at mining company in Zimbabwe, 2010: a cross-sectional study. Pan Afr. Med. J..

[61] Aderaw Z., Engdaw D., Tadesse T. (2011). Determinants of occupational injury : a case control study among textile factory workers in amhara regional state , Ethiopia. J. Trop. Med..

[62] Ajith M.M., Ghosh A.K. (2019). Comparison of parameters for likelihood and severities of injuries in artisanal and small-scale mining (ASM). Saf. Sci..

[63] Mantey J., Nimo F.O., Nyarko K.B., Aubynn A. (2017). Operational dynamics o f ‘Galamsey’ within eleven selected districts of western region of Ghana. J. Min. Environ..

[64] Owusu-Nimo F., Mantey J., Nyarko K.B., Aubynn A. (2018). Spatial distribution patterns of illegal artisanal small scale gold mining (Galamsey) operations in Ghana : a focus on the Western Region. Heliyon.

[65] Laflamme L., Menckel E. (1995). Aging and occupational accidents a review of the literature of the last three decades. Saf. Sci..

[66] Stojadinović S., Svrkota I., Petrović D., Denić M., Pantović R., Milić V. (2012). Mining injuries in Serbian underground coal mines–A 10-year study. Injury.

[67] Calys-tagoe B.N.L., Ovadje L., Clarke E., Basu N. (2015). Injury profiles associated with artisanal and small-scale gold mining in tarkwa , Ghana. Int. J. Environ. Res. Publ. Health.

[68] Sawacha E., Naoum S., Fong D. (1999). Factors affecting safety performance on construction sites. Int. J. Proj. Manag..

[69] Eshun P.A., Okyere E. (2017). Assessment of the challenges in policy implementation in the small scale gold mining sector in Ghana – a case study. Ghana Min. J..

[70] Ghana Business Regulatory Reforms (2012). https://bcp.gov.gh/new/search_detail.php?indexes_id=Mzk2MQ==.

[71] Stephens D.K. (2016). https://www.semanticscholar.org/paper/An-assessment-of-occupational-health-and-safety-in-Stephens/5b68ce6ea991be827dc4198120035a1592f3a7bb.

[72] Leung A.M.R., Leilanie J., Lu D.P. (2016). Environmental health and safety hazards of indigenous small-scale gold mining using cyanidation in the Philippines. Environ. Health Insights.

[73] Debrah A.A., Watson I., Quansah D.P.O. (2014). Comparison between artisanal and small-scale mining in Ghana and South Africa the regional frameworks: yaoundé Vision and the MMSD in the AMV. J. South. African Inst. Min. Metall..

