# Supplementary material for: Socio-demographic effects on role assignment and associated occupational health and safety issues in artisanal and small-scale gold mining in Amansie Central District, Ghana
Source: Heliyon. 2023 Feb 14;9(3):e13741. doi: 10.1016/j.heliyon.2023.e13741 (PMC9976322; doi:10.1016/j.heliyon.2023.e13741)
Supplement: Multimedia component 1 [file mmc1.docx]

Appendix 1: Questionnaire

**The influence of Socio-demographic characteristics on the assignment of roles and its associated Occupational health and safety issues in the Artisanal and Small-scale Gold Mining: The case of Amansie central district, Ghana**

Dear Respondent,

Please, a study is being conducted on the above topic, and would be very grateful if you could respond to the questions developed below. Please, your identity would by no means be revealed in any form; therefore, be at liberty to complete the questionnaire with independent and objective judgment. Information provided shall be treated strictly confidential and for academic purposes only.

Thank you.

**Instructions:** Please tick [ ] the box that best represents your view or state where appropriate.

**SECTION A: Socio-demographic of Respondents**

This section of the questionnaire refers to the background or biographical information. Although the researchers are aware of the sensitivity of the questions in this section, the information provided would allow comparison of groups of respondents. Once again it is assured that your response would remain anonymous.

1. Age group: a. 18 – 25 [ ] b. 26 – 30 [ ] c. 31 – 35 [ ] d. 36 and above [ ]
2. Gender: a. Male [ ] b. Female [ ]

3. Years of experience in the occupation a. 1 year [ ] b. 1-4 years [ ] c. 5-9 years [ ] d. 10 years and over [ ]

4. Educational background a. Junior High School [ ] b. Senior High School [ ] c. Technical and Vocational School [ ] d. Tertiary [ ] e. None [ ]

5. How long have you been involved in ASGM activities? a. less than a year [ ] b. 1 – 4 [ ] c. 5 – 9 [ ] d. 10 and over [ ]

6. How long have you worked in the gold mining industry in general? a. less than a year [ ] b. 1 – 4 [ ] c. 5 – 9 [ ] d. 10 and over [ ]

7. Have you always worked in ASGM? a. Yes [ ] b. No [ ] c. I don’t know [ ]

8. If No, what type of work did you do previously? a. Farming [ ] b. Petty Trading [ ] c. Clerical [ ] d. Driving [ ] e. Other [ ]

9. What are some of the reasons why you work as an ASGM today? a. To be financially well off [ ] b. To support family budget & expenses [ ] c. To have a good family image [ ] d. To be treasured [ ]

**SECTION B:** This section generally comprises questions on (i) the Socio-demographic Characteristics of respondents and their influence on the assignment of roles in ASGM; (ii) the influence of socio-demographic characteristics of respondents on the reasons for engaging in ASGM; (iii) Occupational Health and Safety hazards associated with ASGM and (iv) Reasons for the refusal to use PPEs in ASGM.

10. What type of work do you do? a. Blastman [ ] b. Chiseller [ ] c. Grinder [ ] d. Carrier [ ] e. Washer [ ]

11. Have you always done this same work? a. Yes [ ] b. No [ ]

12. If No, what different capacity were you involved in previously? a. Site Messenger [ ] b. Site Food Vendor [ ] c. On & Off site Driver [ ] d. Other [ ]

13. Do you know of any occupational health hazards that are associated with mining? a. Yes [ ] b. No [ ]

14. If Yes, can you name what you know? a. Rock fall [ ] b. Skin splashes with cyanide, psychophysical impairment [ ] c. malaria and dengue fever [ ] d. shoulder disorder [ ]

15. Have you heard of personal protective equipment (PPE)? a. Yes [ ] b. No [ ] c. Can’t remember [ ]

16. If Yes, what is it used for? a. Don’t know [ ] b. Washing [ ] c. Sampling [ ] d. Protection

[ ]

17. Have you ever used personal protective equipment? a. Yes [ ] b. No [ ] c. Don’t remember [ ]

18. Do you use personal protective equipment now at your workplace? a. Yes [ ] b. No [ ]

19. If No, why don’t you use personal protective equipment? a. Unavailability [ ] b. High cost [ ] c. Waste time [ ] d. Uncomfortable using PPE [ ]

20. Have you or any of your colleagues been arrested for not using personal protective equipment before? a. Yes [ ] b. No [ ] c. Nobody checks this [ ]

21. Are personal protective equipment readily available on the local market? a. Yes [ ] b. No [ ] c. I don’t know [ ]

22. How much does PPE (e.g., safety helmet, boot, overall suit, gloves, goggles, respirators, etc. cost on the local market? a. Gh100-200 [ ] b. 250-300 [ ] c. 350-400 [ ] d. 450-500 [ ] e. 550-600 [ ] f. 650-700 [ ]

23. What would you say about the price of PPE? a. Very moderate [ ] b. Moderate [ ] c. Cheap [ ] d. Expensive [ ]

24. How often do you buy personal protective equipment for use at the workplace? a. Monthly [ ] b. As and when it gets worn out [ ] c. Yearly [ ]

25. Do you enjoy using personal protective equipment? a. Yes [ ] b. No [ ]

25b. If yes, why? a. Very Protective [ ] b. It's cheap [ ] c. It's replaceable [ ] d. It gets me on-site [ ] e. It sets a good example [ ]

25c. If No, why? a. Ignorant [ ] b. Poor enforcement [ ] c. Overconfidence [ ] d. Inconvenience [ ] e. Poor PPE design [ ]

26. What kinds of injuries/accidents are common in this ASGM? a. fall/slip/trip from height [ ] b. being struck by a metallic object [ ] c. being struck a by rock [ ] d. vehicle rollover [ ] e. Poor PPE design [ ] f. being hit by a moving object [ ]

27. Have you been experiencing bodily and chest pains, difficulty breathing, and coughing? a. Yes [ ] b. No [ ]

28. When was the last time you experienced this condition? a. Daily [ ] b. A year ago [ ] c. Can't remember [ ]

29. Where do you seek treatment any time you experience this condition? a. Private Clinic [ ] b. Public Clinic [ ] c. Prayer Camp [ ] d. Shrine [ ] e. Self-Medication and Local Herbs [ ]

**THANK YOU FOR ANSWERING THE QUESTIONS**

Dear Sir/Madam,

Please, a study is being conducted on the above topic and would be very grateful if you could respond to the interview questions developed below. Please, your identity would by no means be revealed in any form; therefore, be at liberty to respond to the interview questions with independent and objective judgment. Information provided shall be treated strictly confidential and for academic purposes only.

Thank you.

**Interview questions for Ghana’s Mineral Commission (MinCom)**

1. Sir/Madam can you please tell us the roles played by the MinCom in the safety and health of ASGM undertakings?
2. Does MinCom offer any safety pieces of training, education, etc. for ASGM?

**Interview questions for Ghana’s Environmental Protection Agency (EPA)**

1. Sir/Madam can you please tell us the roles played by the EPA in the safety and health of ASGM undertakings?
2. Does the EPA offer any safety pieces of training, education, etc. for ASGM?

**THANK YOU FOR ANSWERING THE QUESTIONS**
